# Hierarchically porous monoliths based on low-valence transition metal (Cu, Co, Mn) oxides: gelation and phase separation

**DOI:** 10.1093/nsr/nwaa103

**Published:** 2020-05-27

**Authors:** Xuanming Lu, Kazuyoshi Kanamori, Kazuki Nakanishi

**Affiliations:** Department of Chemistry, Graduate School of Science, Kyoto University, Kyoto 606-8502, Japan; Department of Chemistry, Graduate School of Science, Kyoto University, Kyoto 606-8502, Japan; Institute for Integrated Cell-Material Sciences, Kyoto University Institute for Advanced Study, Kyoto University, Kyoto 606-8501, Japan; Division of Materials Research, Institute of Materials and Systems for Sustainability, Nagoya University, Nagoya 464-8601, Japan

**Keywords:** hierarchically porous monolith, low-valence transition metal oxide, 3D interconnected macropore, sol–gel process, phase separation

## Abstract

Hierarchically porous monoliths based on copper (Cu), cobalt (Co) and manganese (Mn) oxides with three-dimensionally (3D) interconnected macropores and open nanopores were prepared using metal bromides as precursors via a sol–gel process accompanied by phase separation. The difficulty of gelation for low-valence metal cation was overcome by introducing a highly electronegative Br atom near to the metal atom to control the rates of hydrolysis and polycondensation. The 3D interconnected macropores were obtained using appropriate polymers to induce phase separation. The domain sizes of macropores and skeletons can be controlled by reaction parameters such as concentration and/or average molecular weight of polymers, and the amount of hydrochloric acid. The crystalline metal oxide monoliths with their 3D interconnected macroporous structure preserved were obtained after heat treatment in air.

## Teaser

With respect to the difficulty of gelation for low-valence metal oxides, a new route to prepare monolithic gels and hierarchically porous monoliths based on copper, cobalt and manganese oxides is suggested.

## INTRODUCTION

**Scheme 1.**
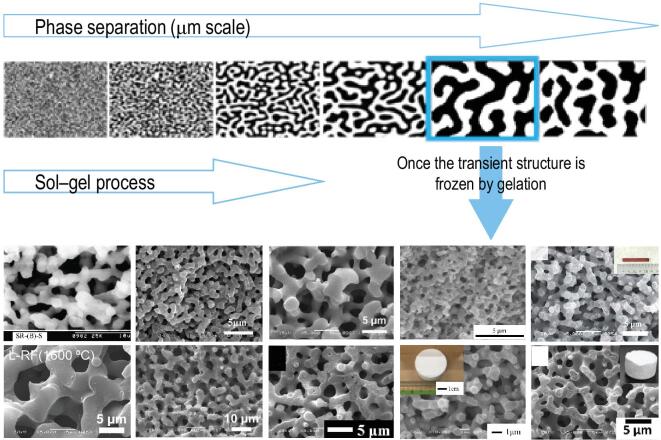
Synthetic strategy based on sol–gel process accompanied by phase separation. From left to right, first row: SiO_2_ [2] (Copyright 2008 Elsevier), Al_2_O_3_ [18] (Copyright 2007 American Chemical Society), TiO_2_ [3] (Copyright 2011 John Wiley and Sons), ZrO_2_ [19] (Copyright 2008 American Chemical Society) and Fe_2_O_3_ [12] (Copyright 2018 Royal Society of Chemistry); second row: carbon [7] (Copyright 2016 Elsevier), LiFePO_4_ [5] (Copyright 2011 American Chemical Society), ZrP_2_O_7_ [10] (Copyright 2014 Royal Society of Chemistry), MgO/niobium phosphate [13] (Copyright 2019 American Chemical Society) and titanium phosphate [23] (Copyright 2015 Royal Society of Chemistry).

Hierarchically porous monoliths, termed ‘HP monoliths’ hereafter, with 3D interconnected macropores and open nanopores have been studied for various applications, such as separation [1–4], electrochemistry [5–9], adsorbence [10–12], catalysis [13] and as support materials for catalyst/enzyme [14,15]. One of the synthetic strategies to prepare HP monoliths is spatial arrest of the transient state in the process of spinodal decomposition by gelation [16], as illustrated in Scheme 1. In this strategy, the 3D interconnected macropores can be obtained via phase separation, and the open nanopores are tailored by aging/drying of networks formed by the polycondensation reactions. Several kinds of materials, including silica [1,2,4,16], metal oxides [3,12,17–21], carbon [7–9] and phosphates [5,10,22,23], have been used successfully for preparation of HP monoliths via a sol–gel process accompanied by phase separation.

In terms of basic metal oxide HP monoliths, there exist two different strategies for preparation with respect to the kind of precursor. The first category of precursor is metal alkoxides (denoted as M(OR)_x_). The first oxide HP monolith prepared via a sol–gel process accompanied by phase separation was SiO_2_ HP monolith [24]. Gelation of SiO_2_ derived from silicon alkoxides has been well studied in sol–gel science, thus preparation of a homogenous SiO_2_ gel is easy. To obtain 3D interconnected macropores, it is necessary to use some water-soluble organic polymer additives, such as poly(ethylene oxide) (PEO), or to modify the solvent composition to control the phase separation [16]. Followed by the success with SiO_2_, TiO_2_ [25,26] and ZrO_2_ [19], HP monoliths have been prepared using this strategy. However, the metal alkoxides generally have faster hydrolysis and polycondensation rates than Si(OR)_4_ because of the relatively high positive partial charge on their central metal atoms. For example, the hydrolysis rate of Ti(OR)_4_ is about 10^5^ times higher than that of Si(OR)_4_ under the same conditions. Needless to say, other tri-valent and di-valent metal alkoxides, which have higher reactivity than Ti(OR)_4_. The high reactivity of many metal alkoxides toward water leads to formation of dispersed precipitates via local quick polycondensation rather than homogeneous gelation with spatially extended network. Thus, it is necessary to suppress the high reaction rate using strong acid [19,25] or complex agents [26] for preparation of a homogenous gel. Under moderate hydrolysis and polycondensation reactions, the morphology developed by phase separation can be better controlled by compositional parameters, e.g. concentrations of polymer, solvent, and catalyst, similar to the case of the SiO_2_-system.

The second category of precursor is ionic salts of metallic elements. Most metal oxide-based monolithic gels and HP monoliths so far reported were prepared via an epoxide-mediated method, as developed by Gash's group [27]. Compared with most metal alkoxides, the inorganic metal salts are less reactive toward hydrolysis and can generally be purchased at lower cost. In the epoxide-mediated method, epoxides act as acid scavengers, raising the solution pH homogenously through an irreversible ring-opening reaction (Eq. 1, e.g. propylene oxide):

**(1):**
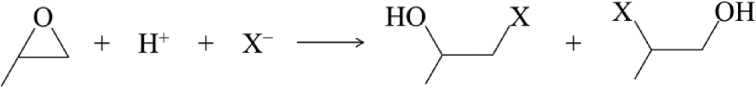


The increase in pH leads to hydrolysis and polycondensation of metal aquo complexes, and thus metal (oxy)hydroxide/oxide gel can be obtained under appropriate conditions. Synthesis of many metal oxide aerogels through the epoxide-mediated method has been reported [28]. Combining the epoxide-mediated method and polymerization-induced spinodal decomposition, Al_2_O_3_, TiO_2_, ZrO_2_, and Fe_2_O_3_ HP monoliths have been successfully prepared [12,17,18,20].

However, it is still a challenge to prepare an oxide gel or HP monolith derived from low-valence metal salts, such as Mg, Mn (II), Co (II), Ni (II), Cu (II), Zn and so on [28], although there are reports of synthesis of some via the epoxide-mediated method [29–31]. We suggest that the difficulty could be because of the low electronegativity of central metal atoms (Table S1). Hydrolysis and polycondensation can be promoted even with atmospheric humidity by nucleophilic reactions [32]. According to the partial-charge model developed by Livage and co-workers, the partial charge on metal atoms in most metal alkoxides is much higher than that on Si in silicon alkoxide [33]. As a result, hydrolysis and polycondensation of metal alkoxides become too fast to form a homogeneous gel with an extended network. Although the epoxide-mediated method greatly extends the window of homogeneous gel formation, it is still difficult to form a homogeneous gel from a solution of low-valence metal salts, as the low-valence metal cations generally have higher positive partial charge than high-valence metal cations. Incorporation of organic molecules or polymers as structure-supporting components to facilitate homogeneous gelation is a common strategy [34–36]. However, the networks of these gels are mainly constructed by interaction between organic supporters and metal-based species rather than polycondensed M−O−M bonds among metal-based species. As a result, the monolithic form and original macroporous structure can hardly be preserved when the organic moieties are oxidatively removed by heat treatment in air.

In the present report, we demonstrate a versatile preparation method of HP monoliths from three kinds of low-valence metal salts. The results and discussion will be divided into three sections: ‘gel formation’, ‘phase separation’, and ‘heat treatment’. In the section on ‘gel formation’, we introduce a way to overcome the difficulty of homogeneous gelation for low-valence metal cations. The starting materials here refer back to the work reported by Hope-Weeks’ group, but the process and mechanism in our work are different [31]. In the section on ‘phase separation’, the effect of starting compositions on 3D interconnected macroporous structure of HP monoliths is discussed. Three factors to control the 3D interconnected macropores are introduced: the concentration of polymer (Cu-system), the average molecular weight of polymer (Co-system) and the amount of hydrochloric acid (Mn-system). In the section on ‘heat treatment’, the preservation of macroporous structure and crystalline phases of the heat-treated samples are presented in detail.

## RESULTS AND DISCUSSION

### Gel formation

Hydrolysis and polycondensation in acidic conditions are determined by the partial negative charge on oxygen. In the present reaction, brominated metal alkoxides are formed by the alkoxylation ring-opened epichlorohydrin (ECH) and metal bromides (MBr_2_, M = Cu, Co, Mn), as expressed in Eq. (2). Here, the highly electronegative Br is expected to modify the distributions of electron density to reduce the negative charge on oxygen upon the formation of brominated metal alkoxide.

**(2):**
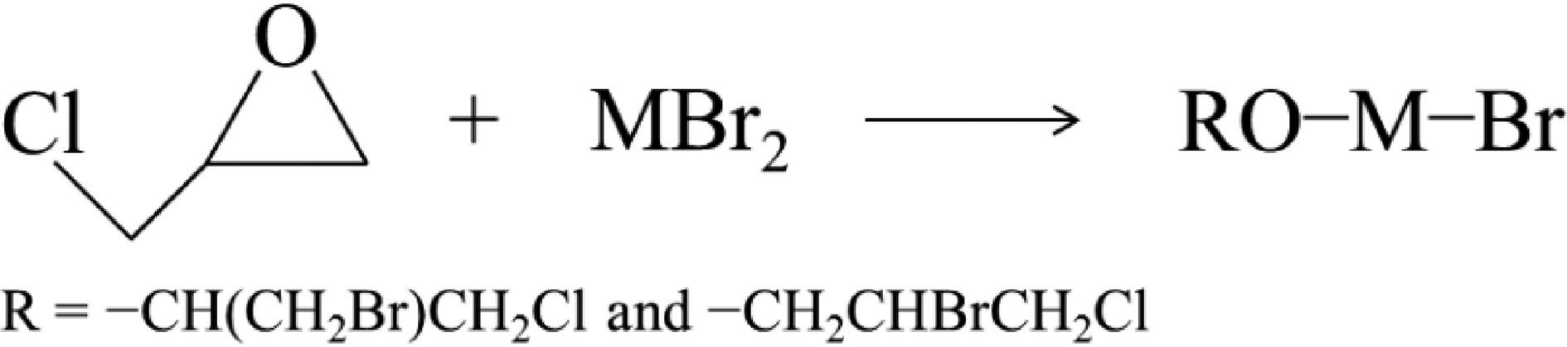


The reaction between MBr_2_ and ECH was carried out in the aprotic solvent, *N, N*-dimethylformamide (DMF), to allow the epoxide to react only with metal salt [37]. Although DMF can dissolve metal salts, it cannot solvate the anions completely [38–40]. The choice of epoxide is based on two considerations: firstly, the reactivity toward metal salt; secondly, the generated alkoxide group, which will have an effect on the hydrolysis rate. The kind of metal salt is chosen based on the following considerations: firstly, the electronegativity of anion; secondly, the difficulty of removal of the anion through post-treatment; thirdly, the solubility in the solvent.

**Figure 1.**
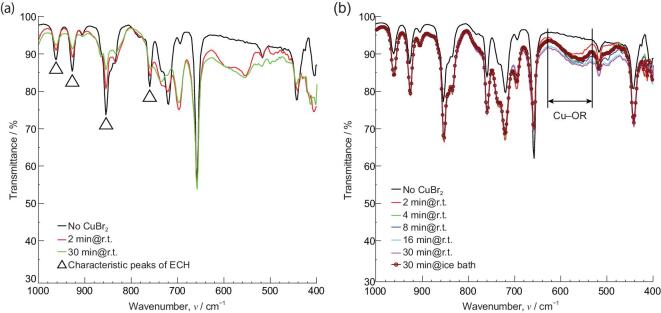
FT-IR spectra of (a) control solution with the stoichiometric ratio of ECH to CuBr_2_ equal to 2:1 over time; (b) solution with practical stoichiometric ratio of ECH to CuBr_2_ over time.

The reactions between ECH and MBr_2_ were qualitatively confirmed by FT-IR spectroscopy. Considering the high mole ratios of ECH to MBr_2_ (n_ECH_/n_Cu_ = ∼6.5, n_ECH_/n_Co (or Mn)_ = ∼8.1) in the typical starting solutions, the control solutions containing ECH and MBr_2_ with mole ratio of 2:1 were characterized also by FT-IR to illustrate the reactions at first. In Cu-system, the absorption of characteristic bands of the epoxy ring (962, 928, 853 and 760 cm^−1^) is decreased over time, but these do not disappear completely within 30 min at room temperature (r.t.) (Fig. 1a) [41]. On the other hand, the emergence of a new absorption band within the range from 628 to 532 cm^−1^ is attributed to the formation of Cu−OR bonding [42–44]. Similar results were obtained in Co- and Mn-systems (Figs S1a and S2a).

Figure 1b shows the FT-IR spectra of solutions with the typical starting composition in Cu-system. The new absorption band of Cu−OR vibration became stronger with the reaction time from 2 to 30 min at r.t. However, the absorption of Cu−OR vibration in the solution that was stirred in an ice-bath for 30 min after the CuBr_2_ dissolved completely, is close to that in the solution with reacting for 4 min at r.t. This means that the ring-opening reaction can be slowed down (or stopped) effectively by cooling in the ice-bath. Considering all the results above, it can be concluded that ECH reacts with CuBr_2_ via a ring-opening reaction to form alkoxide groups, and this reaction can be slowed down (or stopped) in an ice-cooled condition. It can be reasonably deduced that brominated copper alkoxides (CuBr_x_(OR)_y_) with Br ligands and OR groups were produced in the starting solution from incomplete alkoxylation of Cu by the ring-opened product of ECH. In addition, it implies that the relative numbers of Br ligand and OR group can be adjusted by controlling the reaction process via reaction time and temperature, and thus the reactivity of brominated metal alkoxides can be controlled. The use of brominated precursor allows preparation of a homogeneous composite oxide gel, where moderate rates of hydrolysis and polycondensation of the precursors are essential. There are some differences of absorption bands attributed to M^′^−OR (M^′^ = Co, Mn) vibration with varying conditions and reaction times in Co- and Mn-systems. The absorption bands attributed to M^′^−OR show negligible changes with the reaction time (Figs S1b and S2b). The characteristic absorption bands of the epoxy ring decrease after mixing the MBr_2_ and ECH, but show a little change with the reaction time from 2 to 30 min at r.t. (Figs S1 and S2). This indicates that the reaction equilibrium between M’Br_2_ and ECH is almost reached within 2 min at r.t. Nevertheless, it can be deduced that the brominated metal alkoxides with Br ligands and OR groups were produced also in Co- and Mn-systems.

After formation of Br−M−OR intermediates, subsequent processes may be essentially similar to the hydrolysis and polycondensation of silicon alkoxides, which have been well investigated. The presence of the highly electronegative Br ligand, causes the electron density on the oxygen atom in alkoxy ligand to decrease. At the same time, bulky halogenated propyl ligand causes steric hindrance toward hydrolysis, thus slowing the hydrolysis rate. On the other hand, the polycondensation between M−OH is also slowed because of the presence of the Br ligand. As a result, a monolithic gel consisting of the M−O−M network can be prepared by this method.

### Phase separation

The phase separation tendency can be estimated qualitatively by the Flory–Huggins equation (Eq. 3):

(3) \begin{equation*} {\rm{\Delta }}G \propto RT\left( {\frac{{{{\Phi } _{\rm{g}}}}}{{{P_{\rm{g}}}}}\ln {\Phi_{\rm g}} + \frac{{{\Phi _{\rm{s}}}}}{{{P_{\rm{s}}}}}\ln {\Phi _{\rm{s}}} + \chi {\Phi _{\rm{g}}}{\Phi _{\rm{s}}}} \right), \end{equation*}

where Δ*G* is the Gibbs free energy change of mixing; *R* is the gas constant; *T* is the temperature; ${\Phi}$ is the volume fraction; *P* is the degree of polymerization; g and s express gel-rich phase and solvent-rich phase, respectively; *χ* is the interaction parameter between components in gel-rich and solvent-rich phases, which describes the compatibility between two components [45,46]. The former two terms in the bracket express the entropic contribution, and the last term expresses the enthalpic contribution. As ${\Phi}$ denotes the fraction, the entropic term is always negative. The enthalpic term can be negative or positive depending on *χ*. In the polymerizing system, the degree of polymerization in gel-rich phase, *P*_g_ will increase with time, making the entropic term less negative. Under conditions where *χ* is positive, when the absolute value of the entropic term becomes small enough to make Δ*G* positive, the system becomes unstable, and the driving force of phase separation arises. In addition, other parameters, such as volume fraction, ${\Phi}$, and interaction parameter, *χ* also have effects on the phase separation tendency. Here, we present how to control the phase-separated morphology by different experimental parameters based on the Flory–Huggins equation in the next three systems, respectively.

#### Cu-system (effect of concentration of polymers)

**Figure 2.**
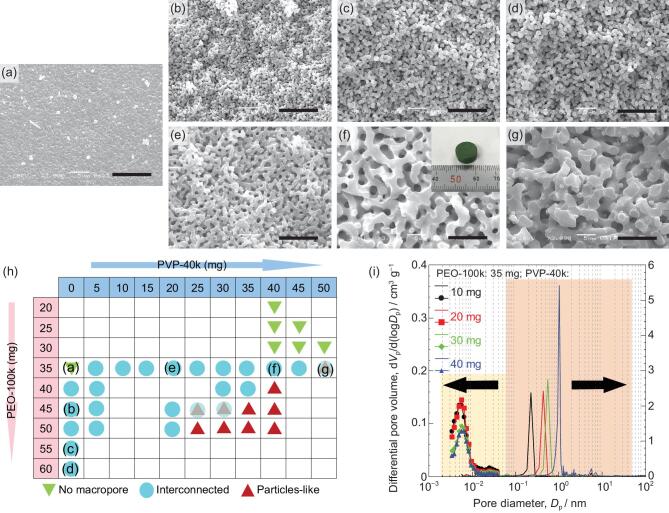
(a–g) SEM images of Cu-based as-dried samples prepared with various concentrations of PVP-40k and PEO-100k; the inset in (f) is the appearance of the Cu-based as-dried sample. Scale bars: 10 μm. (h) Dependence of resultant samples morphology on starting polymer composition. (i) Pore size distributions of Cu-based as-dried samples prepared with varying concentration of PVP-40k. The macropore size distribution was obtained by mercury intrusion porosimetry, and the mesopore size distribution was obtained by nitrogen adsorption–desorption measurement. The adsorption–desorption isotherms are shown in Fig. S3.

In most cases in our previous works, poly(ethylene oxide) (PEO) was employed to induce phase separation, and it was preferentially distributed to the solvent-rich phase [5,10,12,23]. As shown in the SEM images, the morphology of Cu-based as-dried gels changed from no macropores to 3D interconnected macropores when the amount of PEO-100k (*M*_v_ = 100 000 Da) increased from 35 to 60 mg (Fig. 2a–d). This change can be interpreted as follows: with an increase of starting PEO concentration, the volume fraction of PEO-solvent-rich phase increases, accompanied by an increase in phase separation tendency. The sizes of macropores and skeletons, however, depended weakly on the PEO concentration and it was difficult to obtain coarser domain structures by further increasing PEO concentration because of its limited solubility in DMF. For the purpose of controlling the sizes of macropores and skeletons over a broader range, additional polymer, polyvinylpyrrolidone (PVP-40k, *M*_v_ = 40 000 Da), was employed [5,23]. As the amount of PVP was increased from 0 to 50 mg with a fixed amount (35 mg) of PEO, the morphology changed from no macropores to 3D interconnected macropores (Fig. 2e–g). When the amount of PVP increased to 50 mg, small particle-like domains were sparsely found, possibly from secondary phase separation. The phase-separated morphology was observed when the amount of PVP was increased to 5 mg with a fixed amount of PEO and the skeletons became coarser with an increase of PVP. The attractive interaction between PVP and Cu-based oligomers makes them dominant constituents in the gel-rich phase, while PEO and solvents mainly constitute the solvent-rich phase. The increased PVP thus enhanced the phase separation tendency (*χ* was increased), resulting in coarsened morphology of interconnected macropores and skeletons. The morphologies of other samples prepared with varied amounts of PEO and PVP are summarized in Fig. 2h. According to the current results, the compositional ranges favorable for phase-separated (interconnected macroporous) morphology have been confirmed. For example, phase separation can hardly be induced when the amount of PEO is less than 30 mg; with an increase of PEO, the variable range of PVP (for interconnected macropores) becomes narrow. It thus becomes possible to prepare the desired sizes of macropores and skeletons with appropriately selected concentrations of PEO and PVP. The macropore sizes of the samples prepared with 10, 20, 30 and 40 mg of PVP are 0.23, 0.43, 0.55 and 1.02 μm, respectively, and have narrow distributions (Fig. 2i). Meanwhile, the mesopore sizes of all four samples are around 5 nm, which is affected negligibly by the amount of PVP, but mostly determined by gelation and drying processes. Therefore, the sizes of macropores and mesopores can be controlled independently. The specific surface areas of the samples prepared with 10, 20, 30 and 40 mg of PVP, calculated with the Brunauer–Emmett–Teller (BET) method were 61, 58, 38 and 30 m^2^ g^−1^, respectively. Although the median mesopore size showed a negligible change with PVP concentration, the volumes of micropores and mesopores depended inversely on the PVP concentration.

#### Co-system (effect of average molecular weight of polymer)

**Figure 3.**
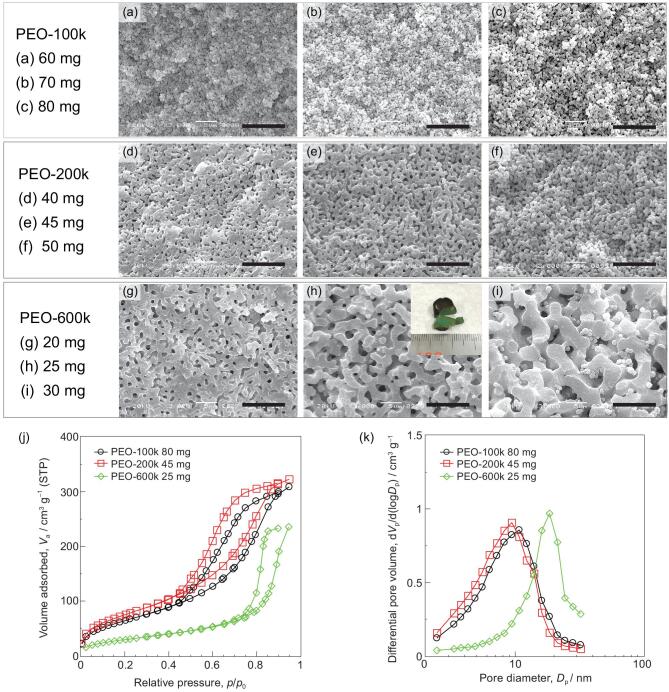
SEM images of Co-based as-dried samples prepared with various amounts and average molecule weights of PEO. PEO-100k: (a) 60 mg, (b) 70 mg, (c) 80 mg; PEO-200k: (d) 40 mg, (e) 45 mg, (f) 50 mg; PEO-600k: (g) 20 mg, (h) 25 mg, (i) 30 mg; PVP-40k: 50 mg for all samples. The inset in (h) is the appearance of the Co-based as-dried sample. The brown color in the surface is because the sample was oxidized into CoO(OH) in air. Scale bars: 10 μm. (j) Nitrogen adsorption–desorption isotherms and (k) mesopore size distributions of the Co-based as-dried samples prepared with different PEO.

In contrast to the Cu-system, the phase separation tendency in the Co-system is relatively weak at any possible concentrations of PEO-100k and PVP-40k. In other words, the domain sizes of gel-rich phase and solvent-rich phase can hardly be controlled in this condition (Fig. 3a–c). In the Co-system, PEO is also considered to be preferentially distributed to the solvent-rich phase. Phase separation is driven by entropy loss from the polymerization of cobalt species and thus depends directly on the molecular weight of respective constituents [16]. The phase separation took place with smaller amounts of PEO-200k (*M*_v_ = 200 000 Da) compared with PEO-100k, as shown in Fig. 3d, and the gels prepared with PEO-200k had coarser morphology than that of gels prepared with PEO-100k (Fig. 3e). However, the phase separation was not further enhanced when the amount of PEO-200k increased to 50 mg (Fig. 3f). Substantially high viscosity of the reaction solution is considered to have suppressed the coarsening of phase-separated structure before gelation [47]. Gels with still coarser morphology were obtained using PEO with average molecular weight PEO-600k (*M*_v_ = 600 000 Da) (Fig. 3g–i). It was found that smaller amounts of PEO were required to obtain a phase-separated structure when PEO with higher molecular weight was employed. Besides, the morphology of the samples was sensitive to the amount of PEO-600k. As shown in the SEM images, sizes of both macropores and skeletons were increased as the amount of PEO-600k changed from 20, to 25 and 30 mg. These results indicate that PEO with higher molecular weight gives a higher phase separation tendency (as *P_s_* is increased), and thus can induce phase separation at a lower concentration. Therefore, the 3D interconnected macropores in the Co-system can be controlled by concentration and average molecular weights of PEO. This will be similarly applicable to other systems in which PEO and metal-based oligomers have weakly attractive interactions. The nitrogen adsorption isotherms show an uptake at low relative pressure indicative of high specific surface area (Fig. 3j). The specific surface areas calculated with the BET method were 244 (PEO-100k: 80 mg), 284 (PEO-200k: 45 mg) and 112 (PEO-600k: 25 mg) m^2^ g^−1^, respectively, and corresponded roughly to the height of the uptakes. Similar mesopore size distributions, which were calculated with the Barrett–Joyner–Halenda (BJH) method, are observed in the samples prepared by PEO-100k and PEO-200k, while the pore size distribution shifts to the larger region in the sample prepared by PEO-600k (Fig. 3k).

#### Mn-system (effect of amount of hydrochloric acid solution)

**Figure 4.**
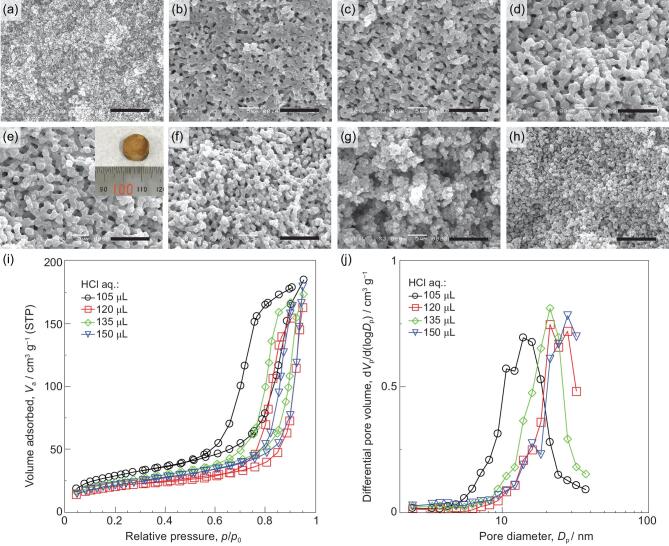
SEM images of Mn-based as-dried samples prepared with various amounts of HCl aq.: (a) 75 μL, (b) 90 μL, (c) 105 μL, (d) 120 μL, (e) 135 μL, (f) 150 μL, (g) 165 μL, (h) 180 μL; the inset in (e) is the appearance of the Mn-based as-dried sample. Scale bars: 10 μm. (i) Nitrogen adsorption–desorption isotherms and (j) mesopore size distributions of the Mn-based as-dried samples prepared with varied amounts of HCl aq. PEO-100k: 40 mg, PVP-40k: 60 mg for all samples.

The morphology with pores in the micrometer scale is a result of the competitive processes of sol–gel transition and phase separation. Any factor affecting sol–gel transition, such as temperature, catalyst, concentration, and those affecting phase separation, such as polymerization, volume fraction of each phase, compatibility between constituents, will determine the final morphology of the sample, which has been well investigated in a silica system by Nakanishi [16]. This indicates that the 3D interconnected macropores can be controlled not only by additive polymers, but also by other parameters. Figure 4 shows SEM images of Mn-based as-dried gels prepared (with 40 mg of PEO-100k and 60 mg of PVP-40k) with varying amounts of diluted aqueous hydrochloric acid (HCl aq.) from 75 to 180 μL. No 3D interconnected macropores were observed in the sample prepared by 75 μL of HCl aq. (Fig. 4a). The 3D interconnected morphology was developed and became coarser when the amount of HCl aq. increased from 90 to 135 μL (Fig. 4b–e) and then it became finer when HCl aq. was further increased from 135 to 150 μL (Fig. 4f). Clearly, the volume fraction of macropores increased monotonously with HCl aq. However, the phase separation tendency only moderately depended on the HCl aq. amount passing through a maximum around 120–135 μL. On the other hand, increase of HCl aq. promoted hydrolysis and polycondensation of Mn-based species and thus accelerated the sol–gel transition. The finer phase-separated structure in the sample prepared with 150 μL of HCl aq. can be attributed to gelation occurring at the earlier stage of phase separation, preventing the phase-separated structures from coarsening. When the amounts of HCl aq. were further increased to 165 and 180 μL, gelation occurred within a few seconds. The phase-separated morphology became uncontrollable under conditions of such quick gelation, as there was not enough time for the system to develop a well-defined 3D interconnected structure (Fig. 4g and h). The amount of HCl aq. also affected the pH of the solvent in which wet gels were aged. The nitrogen adsorption isotherms and the corresponding BJH mesopore size distributions shown in Fig. 4i and j confirm that the resultant mesopores became larger, roughly corresponding to the amount of HCl aq. The specific surface areas of the samples prepared with 105, 120, 135 and 150 μL of HCl aq., calculated with the BET method were 84, 50, 65 and 57 m^2^ g^−1^, respectively. The results imply the possibility of using varying amounts of HCl aq. to control the mesopore size.

### Heat treatment

**Figure 5.**
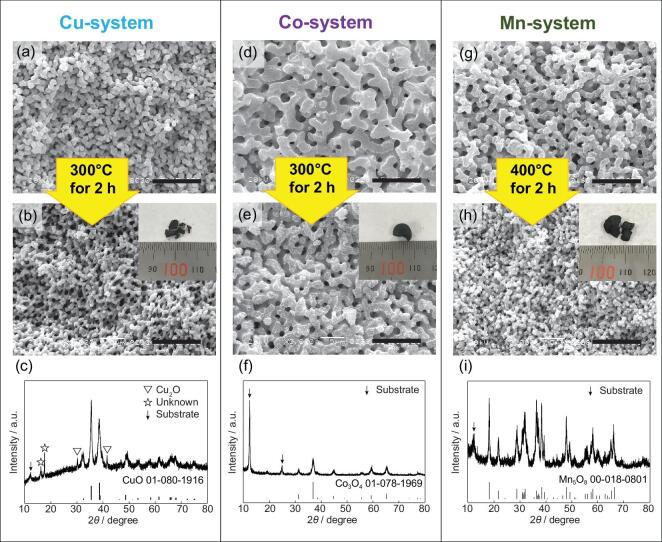
SEM images of the as-dried and heat-treated samples (insets are the appearance of heat-treated samples), and XRD patterns of heat-treated samples in Cu-system (a–c), Co-system (d–f) and Mn-system (g–i), respectively. Scale bars: 10 μm.

All the as-dried samples in three systems are amorphous under X-ray diffractometer (XRD) (may contain fine crystallites). Heat treatment under oxidative conditions is necessary to obtain fully crystalline phases and remove the organic moieties in the skeletons. All the crystalline oxides can be obtained after careful heat treatment in air with their 3D interconnected macroporous structures preserved, although isotropic shrinkages occurred (Fig. 5). In the Cu-system, crystalline CuO was obtained after heat-treatment in air at 300°C for 2 h with some impurity phases under XRD (Fig. 5c). Heat treatment at higher temperature (e.g. 400°C) can remove the impurities, but at the same time destroys the original morphology, resulting in a very fragile heat-treated sample. This may be because other crystals, such as CuBr, Cu and Cu_2_O, form in the process of heating (Fig. S4). Volume changes caused by the multiple/mixed transformation between crystalline structures might lead to the deformation and collapse. In the Co-system, not only the macroporous structure, but also the monolithic form can be preserved after heat-treatment in air at 300°C (Fig. 5d and e). Crystalline Co_3_O_4_ without any impurities was obtained (Fig. 5f). The macroporous structure and monolithic form can be preserved even after heat-treatment at higher temperatures, resulting in Co_3_O_4_ with higher crystallinity. In the Mn-system, crystalline Mn_5_O_8_ retaining the original 3D interconnected macropores was obtained after heat treatment, although isotropic shrinkage and crack formation took place (Fig. 5g–i). In addition, other crystalline phases, such as Mn_2_O_3_ and Mn_3_O_4_ can also be obtained by heat-treatment with varied target temperatures and heating rates (Fig. S5).

## CONCLUSION

Monolithic gels based on low-valence transition metal (Cu, Co, Mn) oxides were prepared successfully through hydrolysis and polycondensation of brominated metal alkoxides, formed by alkoxylation of MBr_2_ by the ring-opened product of ECH in DMF. The Br atom remaining on the alkoxide decreases the partial positive/negative charge on metal/oxygen, and thus decreases the rates of hydrolysis and polycondensation. The open mesopores with narrow distribution in the monoliths are a natural result of sol–gel process. The 3D interconnected macropores are obtained by addition of two kinds of organic polymers, PEO and PVP to induce phase separation. The sizes of phase-separated domains represented by the diameter of macropores and thickness of skeletons can be controlled by the concentration and the average molecular weight of polymers, and the amount of HCl aq. After heat-treatment in air, crystalline metal oxides retaining their 3D interconnected macroporous structures (in all the systems) and monolithic form (in the Co- and Mn-systems) are obtained. This study suggests a way to circumvent the difficulty of homogeneous gelation for low-valence (or less electronegative) metal oxide systems, and provides a novel route to prepare low-valence metal oxide-based gels and HP monoliths.

## METHODS

In a general preparation process, PVP and PEO were dissolved in DMF by heating under stirring. After the polymers dissolved and the solution cooled down to r.t., ECH was added. The mixed solution was transferred to a screw tube bottle containing MBr_2_ (M = Cu, Co, Mn). The solution was stirred in an ice-bath for 30 min after the MBr_2_ dissolved completely. Finally, HCl aqueous solution (35–37%), diluted by H_2_O at a volume ratio of 1:1, was added to the solution in the ice-water bath under stirring, followed by stirring for 5 s. The resulting solution was tightly sealed and placed in an ice-water bath for 1 h to allow gelation. The M-based gel was subsequently aged at 25°C (M = Cu, Co) or 60°C (M = Mn) for 24 h. The gel was dried in a loosely capped bottle at 40°C after solvent exchanges with IPA and *n*-hexane at 40°C for least 8 h each, with each being performed twice. The crystalline samples were heat-treated at varied temperatures for 2 h at a heating rate of 0.25°C min^−1^ to the target temperature in the furnace. For the specific process, please see the Supplementary data.

The structures of the resultant samples in the micrometer range were examined using SEM (JSM-6060S, JEOL, Japan). FT-IR spectra were obtained using a FT-IR spectrometer with attenuated total reflection method (IRTracer-100, Shimadzu Co., Japan). The macropore size distributions were determined using a mercury porosimeter (Autopore IV 9505, Shimadzu Co., Japan). Nitrogen adsorption–desorption isotherms were obtained using a BELSORP-mini II (Microtrac BEL, Japan). The specific surface areas were calculated from an adsorption branch with the BET method. The mesopore size distributions were calculated from the adsorption branch of the isotherm with the BJH method. The crystalline structures of the samples were investigated using an X-ray diffractometer (SmartLab, Rigaku Co., Japan).

## Supplementary Material

nwaa103_Supplemental_File
