# Supplementary material for: Hierarchically porous monoliths based on low-valence transition metal (Cu, Co, Mn) oxides: gelation and phase separation
Source: Natl Sci Rev. 2020 May 27;7(11):1656–66. doi: 10.1093/nsr/nwaa103 (PMC8290958; doi:10.1093/nsr/nwaa103)
Supplement: nwaa103_Supplemental_File [file nwaa103_supplemental_file.docx]

**SUPPORTING INFORMATION**

**Hierarchically porous monoliths based on low-valence transition metal (Cu, Co, Mn) oxides: gelation and phase separation**

Xuanming Lu^1^ · Kazuyoshi Kanamori^1^· Kazuki Nakanishi^2,3 *^

^1^Department of Chemistry, Graduate School of Science, Kyoto University, Kitashirakawa, Sakyo-ku, Kyoto, Kyoto 606-8502, Japan

^2^Institute for Integrated Cell-Material Sciences, Kyoto University Institute for Advanced Study, Kyoto University, Yoshida-Hommachi, Sakyo-ku, Kyoto, Kyoto 606-8501, Japan

^3^Division of Materials Research, Institute of Materials and Systems for Sustainability, Nagoya University, Furo-cho, Chikusa-ku, Nagoya, Aichi 464-8601, Japan

E-mail: dknakanishi@imass.nagoya-u.ac.jp

TEL/FAX: 052-789-3920

**EXPERIMENTAL**

1. Starting materials

Copper (II) bromide (CuBr_2_, >99%), cobalt (II) bromide (CoBr_2_, >99%), manganese (II) bromide (MnBr_2_, >98%), polyvinylpyrrolidone (PVP-40k, *M*_v_ = 40 000 Da), and poly(ethylene oxide)s (PEO-100k, PEO-200k, PEO-600k, *M*_v_ = 100 000, 200 000, 600 000 Da, respectively) were purchased from Sigma-Aldrich Japan. *N,N*-dimethylformamide (DMF, >99.5%), hydrochloric acid (HCl, 35~37%), distilled water (H_2_O), 2-propanol (IPA, >99%), and *n*-hexane (>95%) were purchased from Kishida Chemical Co., Ltd. (Japan). Epichlorohydrin (ECH, >99%) was purchased from Tokyo Chemical Ind. Co., Ltd. (Japan). All the agents were used without further purification.

2. Preparation of Cu-based monolith

Predetermined amounts of PVP-40k and PEO-100k were dissolved in 1 mL of DMF by heating under stirring. After the polymers were dissolved, the solution was cooled down to room temperature, and then 1.5 mL of ECH was added. 3 mmol of CuBr_2_ were dissolved completely in the above mixed solution within 3 min at room temperature, and kept stirred in an ice-bath for 30 min. Finally, the precooled 0.1 mL of diluted HCl aq. was added into the solution in the ice-bath under stirring, followed by stirring for 5 s. The resultant solution was tightly sealed and placed in the ice-bath for 1 h to allow gelation. The gel was subsequently aged at 25°C for 24 h. Solvent exchange was carried out with IPA and *n*-hexane at 40°C for at least 8 h in respective solvents (each for twice). Finally, the gel was dried by slow evaporation in a loosely capped bottle at 40°C.

3. Preparation of Co-based monolith

Predetermined amounts and average molecule weights of PEO and 50 mg of PVP-40k were dissolved in 1.5 mL of DMF by heating under stirring. After the polymers were dissolved, the solution was cooled down to room temperature, and then 2.5 mL of ECH was added. 4 mmol of CoBr_2_ were dissolved completely in the above mixed solution within 3 min at room temperature, and kept stirred in an ice-bath for 30 min. Finally, the precooled 0.15 mL of diluted HCl aq. was added into the solution in the ice-bath under stirring, followed by stirring for 5 s. The resultant solution was tightly sealed and placed in the ice-bath for 1 h to allow gelation. The gel was subsequently aged at 25°C for 24 h. Solvent exchange was carried out with IPA and n-hexane at 40°C for at least 8 h in respective solvents (each for twice). Finally, the gel was dried by slow evaporation in a loosely capped bottle at 40°C.

4. Preparation of Mn-based monolith

40 mg of PEO-100k and 60 mg of PVP-40k were dissolved in 1.5 mL of DMF by heating under stirring. After the polymers were dissolved, the solution was cooled down to room temperature, and then 2.5 mL of ECH was added. 4 mmol of MnBr_2_ were dissolved completely in the above mixed solution within 3 min at room temperature, and kept stirred in an ice-bath for 30 min. Finally, the predetermined amount of precooled diluted HCl aq. was added into the solution in the ice-bath under stirring, followed by stirring for 5 s. The resultant solution was tightly sealed and placed in the ice-bath for 1 h to allow gelation. The gel was subsequently aged at 60°C for 24 h. Solvent exchange was carried out with IPA and n-hexane at 40°C for at least 8 h in respective solvents (each for twice). Finally, the gel was dried by slow evaporation in a loosely capped bottle at 40°C.

**Table S1** Electronegativity (χ_i_) of cations with coordination number (CN) of 4 or 6 *^a^*

| **Cation** | **χ_i_** | **Cation** | **χ_i_** |
| --- | --- | --- | --- |
| Si^4+^ (CN=4) | 2.245 | Mn^2+^ | 1.263 |
| Al^3+^ | 1.513 | Co^2+^ | 1.321 |
| Ti^4+^ | 1.730 | Cu^2+^ | 1.372 |
| Cr^3+^ | 1.587 | Zn^2+^ | 1.336 |
| Fe^3+^ | 1.556 | Y^3+^ | 1.340 |
| Ni^2+^ | 1.367 |  |  |
| Zr^4+^ (CN=4) | 1.743 |  |  |
| Nb^5+^ | 1.862 |  |  |
| Ta^5+^ | 1.925 |  |  |

*^a^* χ_i_ is calculated from eq. $\chi_{i}=0.105n^{*}{\left( {I_{m}}/R \right)^{1/2}}/{r_{i}}+0.863$, where $n^{*}$ is the effective principal quantum number; *R* is Rydberg constant; $r_{i}$ is ionic radii [S1].


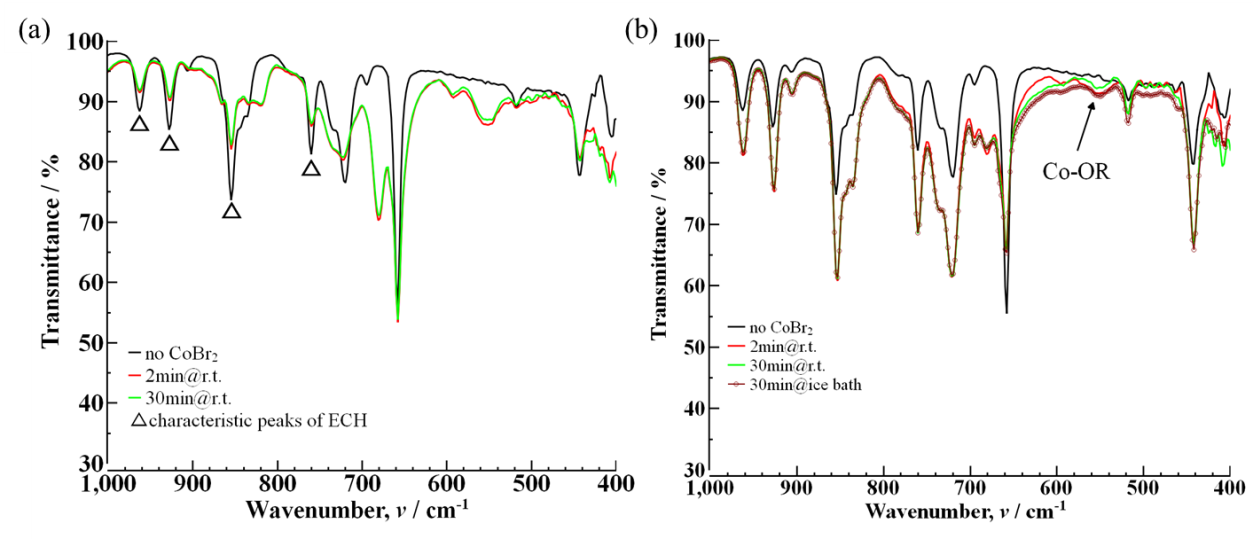


**Figure S1** FT-IR spectra of (a) control solution with the stoichiometric ratio of ECH to CoBr_2_ equal to 2:1 over time; (b) solution with practical stoichiometric ratio of ECH to CoBr_2_ over time.


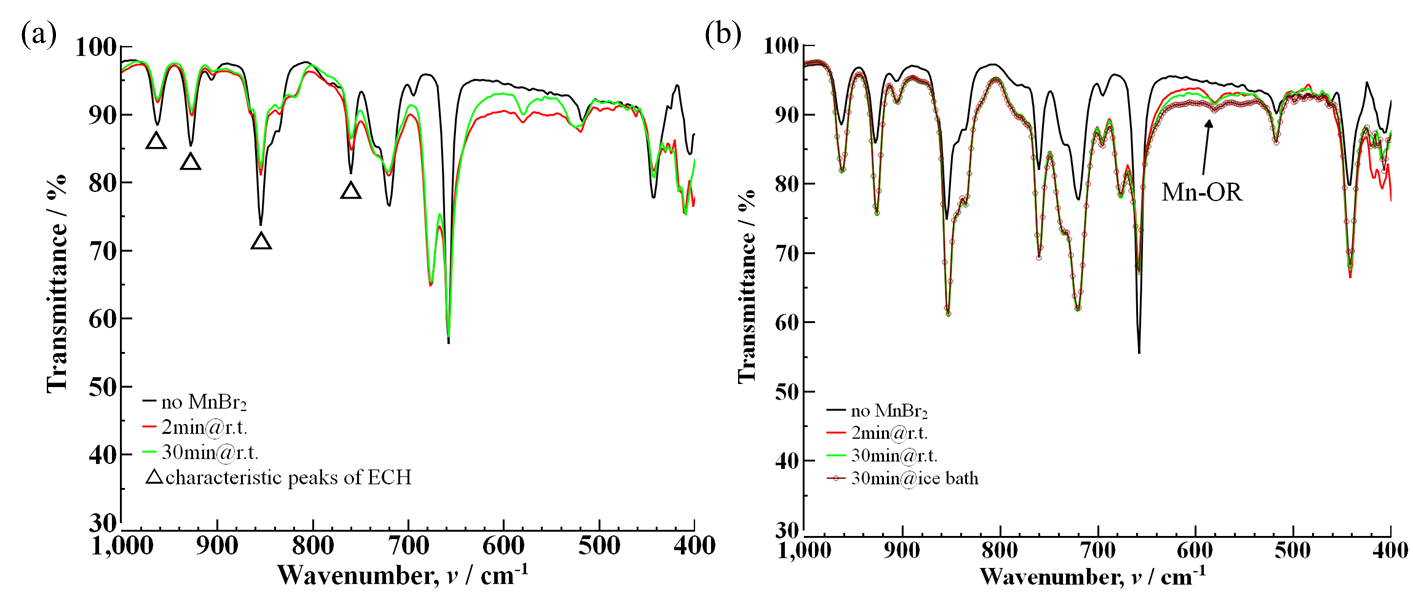


**Figure S2** FT-IR spectra of (a) control solution with the stoichiometric ratio of ECH to MnBr_2_ equal to 2:1 over time; (b) solution with practical stoichiometric ratio of ECH to MnBr_2_ over time.


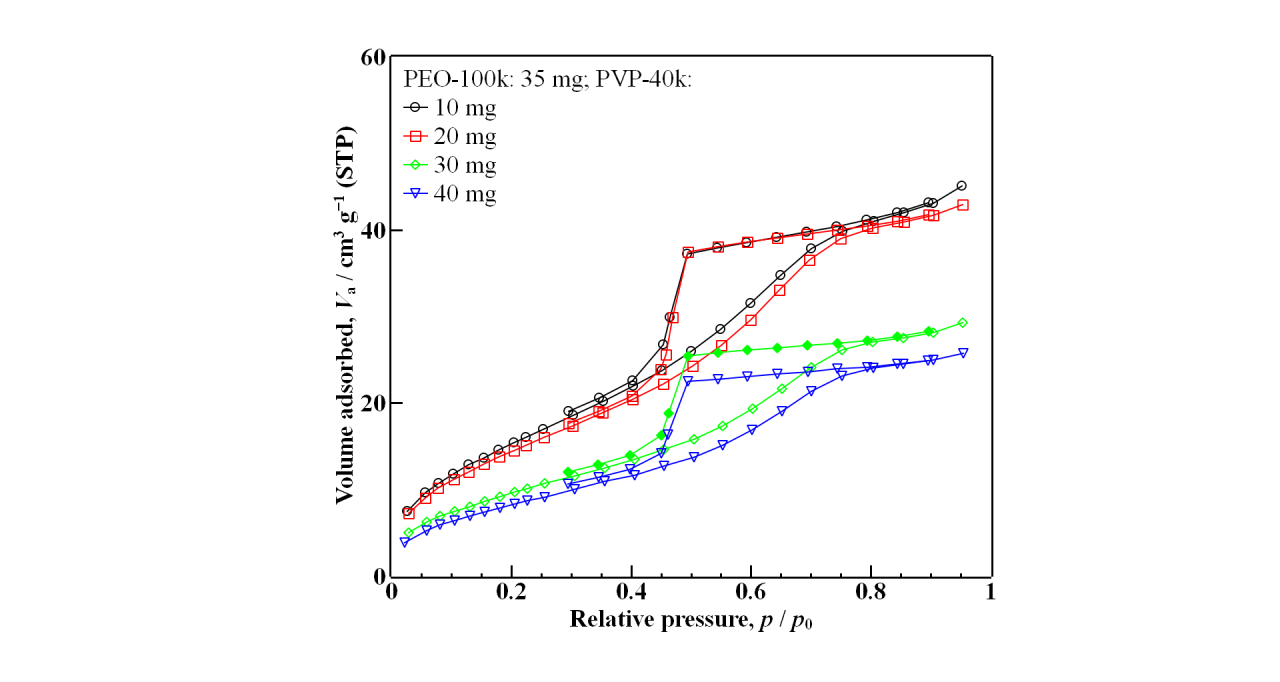


**Figure S3** Nitrogen adsorption isotherms of the Cu-based as-dried samples prepared with varied amount of PVP.


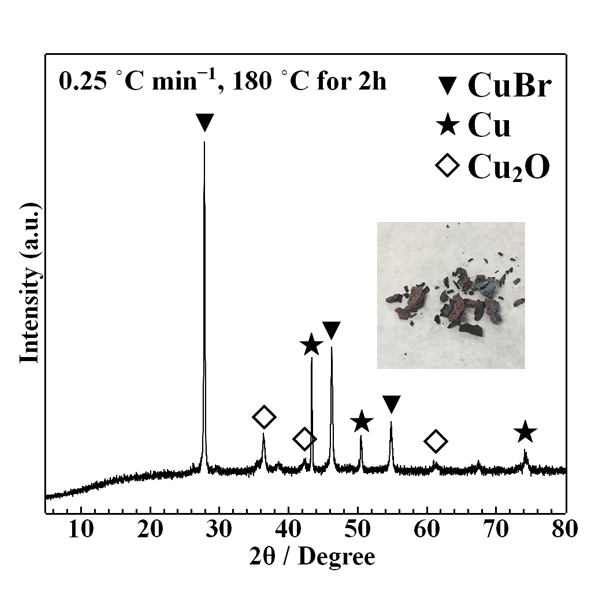


**Figure S4** XRD pattern of samples in Cu-system heat-treated at 180 °C for 2h; the inserted photo is the appearance of the heat-treated sample.


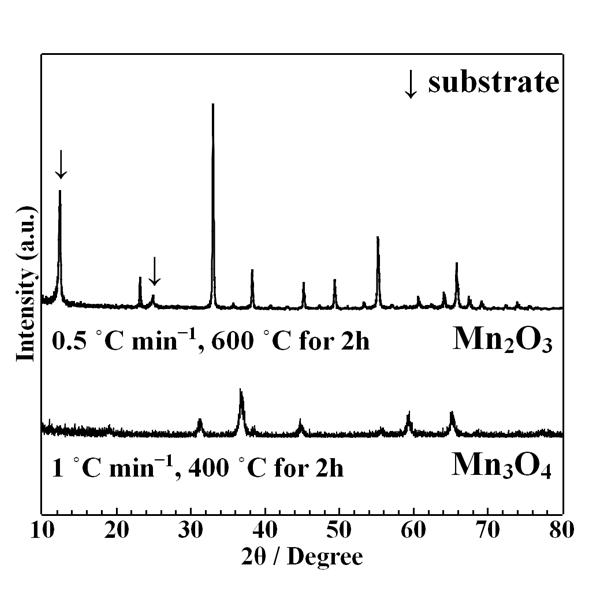


**Figure S5** XRD pattern of manganese oxides heat-treated in varied conditions.

REFERENCES

[S1]. Xue D and Li K. Estimation of electronegativity values of elements in different valence states. *J Phys Chem A* 2006; **110**: 11332-7.
